# Levothyroxine supplementation among individuals with Subclinical Hypothyroidism and Depression | a review

**DOI:** 10.1192/j.eurpsy.2022.1437

**Published:** 2022-09-01

**Authors:** M. Ribeiro, A. Lourenço, M. Lemos, A. Duarte

**Affiliations:** Centro Hospitalar Lisboa Norte, Psychiatry, Lisboa, Portugal

**Keywords:** subclinical hypothyroidism, levothyroxine, Depression

## Abstract

**Introduction:**

Depression is known to be associated with changes in the hypothalamic-pituitary-thyroid axis and the brain is a major target organ for thyroid hormone. Overt hypothyroidism can cause symptoms compatible with depression. However, its relationship with subclinical hypothyroidism (SCH) is not well established.

**Objectives:**

To review the literature regarding the effect of levothyroxine therapy among patients with SCH and coexistent depression.

**Methods:**

We conducted a MEDLINE search using depression, subclinical hypothyroidism and levothyroxine as keywords, selecting studies written in English.

**Results:**

SCH is defined as an elevated thyroid stimulating hormone with normal peripheric hormone levels. The association between SCH and depression is controversial. Some studies indicate that SCH had the same propensity with overt hypothyroidism, while others report that major affective symptoms are not associated with SCH, but are likely due to independent psychiatric diagnoses, which are common in the general population and occur with similar frequency in patients with SCH. Individuals with SCH are recommended to initiate levothyroxine replacement therapy only when their TSH level is above 10 mIU/L or if symptoms are present. There is a lack of evidence supporting the use of levothyroxine therapy to improve mental health outcomes and the majority of meta-analysis do not show relief of affective symptoms after levothyroxine therapy, among individuals with SCH.

**Conclusions:**

Routine screening for depressive symptoms among individuals with SCH is important to prevent morbidity. Nevertheless, there is no evidence enduring levothyroxine supplementation in these cases. Further studies, with larger sample sizes and longer follow-up periods are needed to enlighten the potential benefit of this therapy.

**Disclosure:**

No significant relationships.

